# Spatio-temporal differences in leaf physiology are associated with fire, not drought, in a clonally integrated shrub

**DOI:** 10.1093/aobpla/plab037

**Published:** 2021-06-09

**Authors:** Emily R Wedel, Kimberly O’Keefe, Jesse B Nippert, Braden Hoch, Rory C O’Connor

**Affiliations:** 1 Division of Biology, Kansas State University, 116 Ackert Hall, Manhattan, KS 66502, USA; 2 Department of Botany, University of Wisconsin, Madison, WI 53706, USA; 3 Plant Protection and Weed Control Program, Kansas Department of Agriculture, Manhattan, KS 66502, USA; 4 USDA-Agricultural Research Service, Eastern Oregon Agricultural Research Center, 67826-A Hwy 205, Burns, OR 97720, USA

**Keywords:** Clonal shrubs, *Cornus drummondii*, gas exchange, leaf physiology, shrub encroachment, tallgrass prairie

## Abstract

In highly disturbed environments, clonality facilitates plant survival via resprouting after disturbance, resource sharing among interconnected stems and vegetative reproduction. These traits likely contribute to the encroachment of deep-rooted clonal shrubs in tallgrass prairie. Clonal shrubs have access to deep soil water and are typically thought of as relatively insensitive to environmental variability. However, how leaf physiological traits differ among stems within individual clonal shrubs (hereafter ‘intra-clonal’) in response to extreme environmental variation (i.e. drought or fire) is unclear. Accounting for intra-clonal differences among stems in response to disturbance is needed to more accurately parameterize models that predict the effects of shrub encroachment on ecosystem processes. We assessed intra-clonal leaf-level physiology of the most dominant encroaching shrub in Kansas tallgrass prairie, *Cornus drummondii*, in response to precipitation and fire. We compared leaf gas exchange rates from the periphery to centre within shrub clones during a wet (2015) and extremely dry (2018) year. We also compared leaf physiology between recently burned shrubs (resprouts) with unburned shrubs in 2018. Resprouts had higher gas exchange rates and leaf nitrogen content than unburned shrubs, suggesting increased rates of carbon gain can contribute to recovery after fire. In areas recently burned, resprouts had higher gas exchange rates in the centre of the shrub than the periphery. In unburned areas, leaf physiology remained constant across the growing season within clonal shrubs (2015 and 2018). Results suggest single measurements within a shrub are likely sufficient to parameterize models to understand the effects of shrub encroachment on ecosystem carbon and water cycles, but model parameterization may require additional complexity in the context of fire.

## Teaser

Clonal shrubs are encroaching at an accelerated rate compared to non-clonal shrubs in tallgrass prairie. We compared leaf physiology from the periphery to the centre within the most dominant encroaching shrub in a Kansas tallgrass prairie. We found that leaf physiology does not differ among interconnected stems during a wet or dry year but does differ after fire. Results suggest single measurements within shrub clones are sufficient to parameterize models used to understand the effects of shrub encroachment on ecosystem carbon and water cycles, but models may require additional complexity in the context of fire.

## Introduction

Shrubs are a widespread plant growth form which have evolved in response to multiple drivers of disturbance such as fire, drought and temperature stress (Stutz 1989; Rundel 1991). Götmark *et al.* (2016) estimated that woody shrubs can grow in environments covering approximately 45 % of the terrestrial surface largely because they are successful in harsh climates and frequently disturbed environments where trees do not thrive (e.g. grasslands, tundra, Mediterranean shrublands and semi-arid and arid ecosystems). Shrubs with a multi-stemmed growth form have a number of advantages in disturbed systems compared with single-stemmed shrubs or trees, including increased growth rates and protection from herbivory and wind damage (Peterson and Jones 1997; Rowe and Speck 2005; Götmark *et al.* 2016). In grasslands and savannas, established shrubs recover quickly after disturbances such as fire, drought and/or herbivory via resource sharing and resprouting from protected below-ground, basal or epicormic buds (Lamont *et al.* 2011).

Many shrubs are also clonal, where individuals reproduce asexually via the vegetative production of stems from below-ground buds and rhizomes, which can provide an additional competitive advantage over non-clonal shrubs in frequently disturbed environments. For instance, clonal shrubs are able to spread vegetatively and rapidly resprout after fire or herbivory due to their reserve population of below-ground buds (Ott *et al.* 2019). Clonal shrubs also exhibit physiological integration among connected stems (‘ramets’) which facilitates resource transfer across the entire individual shrub (‘clone’ or ‘genet’) and reduces competition with neighbouring plants. Clonal growth increases the survival and longevity of individuals by providing a stable source of resources to younger or damaged ramets, particularly in environments that experience considerable disturbance and climatic variability (Peterson and Jones 1997; Ratajczak *et al.* 2011; Killian 2012; Nippert *et al.* 2013; Pinno and Wilson 2014; Liu *et al.* 2016). Together, traits associated with clonal growth help mitigate competition for limiting resources, circumvent the challenges of seedling establishment (Hartnett and Bazzaz 1983), and increase the invasiveness of shrubs in disturbed landscapes (Song *et al.* 2013; You *et al.* 2014).

Clonal traits that increase shrub resistance to disturbance may also facilitate the spread of clonal shrubs across herbaceous ecosystems where they are native. Shrub encroachment—the increase in abundance and distribution of shrubs in grasslands and savannas—is occurring worldwide and has been associated with the spread of clonal shrubs within different ecosystems [e.g. *Cornus drummondii* in tallgrass prairie USA (Briggs *et al* 2005; Ratajczak *et al.* 2011); *Dichrostachys cinerea* in lowveld savannas (Wakeling & Bond 2009); *Salix planifolia* and *S. glauca* in alpine tundra (Formica *et al.* 2014); *Larrea tridentata* in south western USA (MacMahon and Wagner 1985)]. Shrub encroachment is broadly driven by changes in climate including increased atmospheric CO_2_ concentrations and altered precipitation regimes (Van Auken 2009; Devine *et al.* 2017), as well as reduced fire frequency and overgrazing associated with land management practices (Briggs *et al.* 2005; Archer *et al.* 2017). Many clonal species exhibit traits that enable their spread in response to these global, regional and local drivers. For example, in North American tallgrass prairie, clonal shrub establishment and growth is limited by frequent fire (burned every 1–3 years; Briggs *et al.* 2005; Ratajczak *et al.* 2014). During longer periods without fire, shrubs are able to establish and form dense canopies via clonal vegetative growth, which reduces light penetration for understory herbaceous species and consequently limits fine-fuel accumulation and the spread of fire through the shrub canopy (Ratajczak *et al.* 2011). Substantial non-structural carbohydrate storage below-ground also allows vigorous resprouting and improves competition with grasses in a post-fire environment (Janicke and Fick 1998; O’Connor *et al.* 2020). Combined, responses to infrequent fire that are associated with shrub clonality create positive feedbacks that can facilitate the conversion of grasslands to shrublands (Ratajczak *et al.* 2011, 2014).

Increased cover of deep-rooted clonal shrubs in grasslands is predicted to shift ecosystem carbon cycling and ecohydrology through increased above-ground biomass and evapotranspiration (Knapp *et al.* 2008; Logan and Brunsell 2015; O’Keefe *et al.* 2020). Given that clonal shrubs are more likely to expand across grasslands and savannas than non-clonal shrubs (Ratajczak *et al.* 2011; Formica *et al.* 2014), understanding how this growth form uses resources and responds to environmental variation will be key for predicting how shrub encroachment may alter carbon and water cycling in the future. There is abundant evidence showing clonal shrubs can translocate resources throughout a clone, and these dynamics can impact the response of a clone to disturbance. For instance, Zhang *et al.* (2002) observed that carbon translocation within the rhizomatous shrub *Hedysarum laeve* can improve growth following defoliation, while Luo *et al.* (2015) showed that *Alhagi sparsifolia* increases the water status of younger ramets by transferring water within a clone. Early work on clonality suggested that resource translocation may be transient; Hartnett and Bazzaz (1983) showed that physiological integration within a common forb (*Solidago canadensis*) decreases with ramet age but that individuals can become ‘reintegrated’ when resources become limiting. Together, these studies indicate that physiological integration, and the resulting impacts on leaf physiological processes, may vary in response to climate and disturbance and may not always be uniform within an individual clone. If this is the case, land-surface models that predict woody plant dynamics may require detailed assessments of physiological differences across ramets within single clones (hereafter ‘intra-clonal’ differences in physiology) to produce more accurate estimates of shrub demography, competition dynamics between shrubs and grasses and resultant shifts in grassland carbon and water cycling.

**Figure 1.**
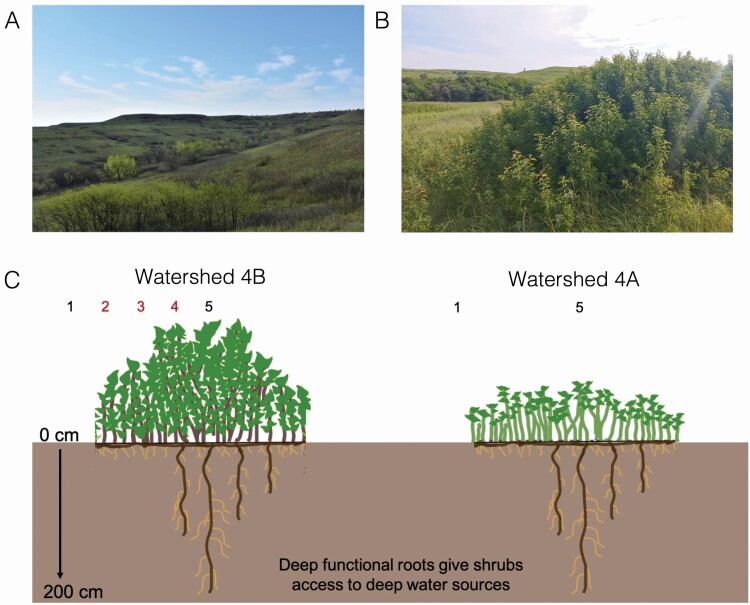
(A) Photo from Konza Prairie Biological Station (KPBS) showing *Cornus drummondii* shrubs scattered across the hilly landscape. KPBS is made up of rolling hills where woody cover is highest in the lowland areas. The brown and green patches are discrete *C. drummondii* shrub clones. (B) Discrete *C. drummondii* shrub clone. Clones grow radially to create discrete groups of stems with dense canopies. Photograph: E. Greg Tooley. (C) Diagram of *C. drummondii* growth form and sampling locations. We sampled shrubs within watershed 4B in 2015 and 2018. Red numbers indicate ramet locations that were only sampled in 2015. In 2018, we sampled resprouting shrubs in watershed 4A that had 100 % above-ground ramet mortality after fire. Shrubs in watershed 4A were burned in April of 2018.

In this study, we examined how leaf-level physiology differs among ramet locations within a major encroaching clonal shrub, *C. drummondii*, in North American tallgrass prairie. *Cornus drummondii* expands radially via rhizomes forming discrete clones where the centre of the shrub is older than the periphery (Fig. 1). Mature *C. drummondii* shrubs resprout rapidly after fire and have deep roots near the centre of the shrub that access deep soil water which can be transferred to juvenile ramets on the shrub periphery (Ratajczak *et al.* 2011). Access to deep and stable water sources and resource transfer among ramets mitigates competition for water with neighbouring grasses and can decouple shrub leaf physiology from typical environmental variability (Ratajczak *et al.* 2011; Nippert *et al.* 2013; Muench *et al.* 2016). While this physiological decoupling has been shown to buffer intraspecific variation among shrubs across the landscape (Nippert *et al.* 2013), how physiological integration impacts leaf-level physiology within a clone, or if the effects of integration on leaf physiology shift in response to more extreme environmental variation such as fire or drought, is unknown. To address this knowledge gap, we asked the following questions: (i) Does leaf-level physiology differ among ramet locations within *C. drummondii* clones? (ii) How does leaf-level physiology within clonal shrubs differ between a wet vs. extremely dry year? and (iii) How does fire affect clonal shrub leaf-level physiology? We compared the leaf-level physiology, including water potential, photosynthetic gas exchange rates, water use efficiency (WUE), nitrogen content and photosynthetic nitrogen use efficiency (PNUE) of *C. drummondii* ramets from the periphery to the centre within shrub clones during a wet (2015) and extremely dry (2018) year. In addition, we compared the leaf-level physiology of *C. drummondii* ramets resprouting after fire (burned in 2018) to shrubs with the same fire frequency (every 4 years) but burned the previous year (burned in 2017). We hypothesized that (i) clonal integration will buffer physiological differences among ramets within a shrub clone resulting in no differences among ramet locations; (ii) deep functional roots provide *C. drummondii* with a stable water source and will reduce differences in photosynthetic gas exchange rates across wet and dry years (Nippert *et al.* 2013); (iii) shrubs resprouting post-fire (hereafter referred to as ‘resprouts’) will have higher gas exchange rates, lower WUE and increased foliar nitrogen (N) content than shrubs burned the previous year (hereafter referred to as ‘unburned’) because fire increases N availability and nutrient use efficiency in infrequently burned grasslands (Ojima *et al.* 1994; McCarron and Knapp 2003). Additionally, shrubs that experience complete mortality of above-ground biomass, or topkill, after fire have an increased root:shoot ratio (Clemente *et al.* 2005; Nolan *et al.* 2014), increasing water and nutrient availability for resprouting ramets and allowing shrubs to maximize carbon gain to recover lost biomass.

## Methods

### Study site

Data were collected at Konza Prairie Biological Station (KPBS; 39°05′N, 96°35′W) located south of Manhattan, KS, USA. KPBS is a 3387 ha native tallgrass prairie characterized by rocky limestone hillslopes. KPBS is divided into watersheds with various burn frequency treatments (1-, 2-, 4-, and 20-year burn intervals) and grazing treatments (bison, cattle or no large mammalian grazers). Frequently burned areas (burned every 1–2 years) are dominated by C_4_ warm season grasses (*Andropogon gerardii*, *Panicum virgatum*, *Sorghastrum nutans* and *Schizachyrium scoparium*). Infrequently burned areas (burned every 4 or more years) are encroached by clonal shrubs (*C. drummondii* and *Rhus glabra*). Long-term mean annual precipitation (1982–2018) is 835 mm with 75 % of precipitation occurring over the growing season (April–September). In 2015 and 2018, growing season precipitation was 747 mm and 516 mm, respectively **[see****Supporting Information—Fig. S1****]**. In 2018, only 30 % of growing season precipitation fell during the sampling period (June–August) and herbaceous biomass was only 25 % of the long-term average (Blair and Nippert 2020). Mean daily temperatures (1982–2018) range from a low of −1.2 °C in January to a high of 26.1 °C in July. In 2018, maximum daily temperatures were 5.6 and 3.6 °C higher than the long-term average for May and June, respectively.

### Study species

*Cornus drummondii* is a deciduous clonal shrub native to eastern Kansas that has undergone encroachment of tallgrass prairie during the past 40 years (Briggs *et al.* 2005). *Cornus drummondii* flowers May–June and sets fruit in July. Shrubs spread laterally via rhizomes, forming new ramets from below-ground buds, and each shrub appears as a discrete group of stems (Fig. 1). Discrete shrubs can grow extremely large and have been reported to cover up to 830 m^2^ (Connell *et al.* 2021). Ramets can grow between 2 and 6 m tall and expand laterally with younger and shorter ramets on the periphery of the clone compared with those in the centre. Fire increases ramet densities and lateral expansion (McCarron and Knapp 2003) as ramets resprout rapidly from basal and below-ground buds.

### Sampling

We sampled mature *C. drummondii* shrubs of varying sizes (14–347 m^2^) in 2015 and 2018 at KPBS. We calculated shrub canopy area using an ellipse area equation by measuring the length of the longest axis and its perpendicular width through each discrete shrub clone. Shrubs were located in the lowlands of two adjacent watersheds (referred to at KPBS as 4A and 4B; 18.84 and 54.5 ha, respectively), each with a 4-year burn frequency. KPBS hosts a long-term ecological research station where prescribed burning occurs during the spring (March–April). Burn history and data legacies from watersheds 4A and 4B date back to 1981 and are publicly available (lter.konza.ksu.edu). Watershed 4B was last burned in 2017 and 4A was last burned in April 2018.

### 2015 sampling

In 2015, we collected leaf-level gas exchange and leaf water potential from six shrubs located in the lowlands of 4B. We sampled five ramets equidistant from the periphery of the clone to the centre (Location 1 = outermost ramet on the periphery and Location 5 = the innermost ramet in the centre; Fig. 1C) on six sampling dates throughout the growing season (June–September; **see****Supporting Information—Table S1**). Instantaneous leaf-level gas exchange measurements of net photosynthetic rate (*A*_net_, µmol CO_2_ m^−2^ s^−1^), transpiration (*E*, mmol H_2_O m^−2^ s^−1^) and stomatal conductance to vapour (*g*_s_, mol H_2_O m^−2^ s^−1^) were measured and intrinsic water use efficiency (iWUE; *A*_net_*/g*_s_) was estimated on one fully expanded, sun-exposed leaf at the five ramet locations within each shrub (*n* = 30) using the Li-6400XT open-system gas analyser (Li-Cor, Inc., Lincoln, NE, USA). Cuvette conditions were set to [reference CO_2_] = 400 µmol CO_2_ mol^−1^, relative humidity = 40–60 %, and photosynthetically active radiation (PAR) = 2000 µmol m^−2^ s^−1^. Leaves were allowed to stabilize to chamber conditions before recording the measurement. Gas exchange measurements were collected between 10:00 h and 15:00 h. This is a similar time window used in other studies on this species (Muench *et al.* 2016; O’Keefe and Nippert 2018; O’Connor *et al.* 2020) and there is no evidence of midday decline in sap flux throughout the growing season (O’Keefe *et al.* 2020). Sampling order was random for each sampling date to minimize variability contributed by diurnal effects.

Predawn (ψ _pd_) and midday (ψ _md_) leaf water potentials were measured on clear, sunny days six times throughout the growing season **[see****Supporting Information—Table S1****]**. Leaves were collected for ψ _pd_ measurements approximately 1 h prior to dawn and leaves used for ψ _md_ measurements were collected at approximately 12:00 h. We collected the youngest, fully expanded leaf and equilibrated the leaf for 1 h in a dark, high [CO_2_] moist plastic bag to ensure stomatal closure. Leaf water potential was measured using a Scholander pressure chamber (PMS Instrument Company, Albany, OR, USA).

### 2018 sampling

In 2018, we assessed leaf-level physiological responses to fire. We measured leaf physiological traits on 20 shrubs located in 4B (unburned) and 20 shrubs located in 4A (resprouts). For the resprouts, we selected individuals that experienced 100 % above-ground ramet mortality following fire. We measured leaf-level gas exchange and iWUE using the same methods as in 2015 on one ramet on the periphery and one ramet in the centre of each shrub clone during four sampling dates throughout the growing season (June–August; **see****Supporting Information—Table S1**). Samples from resprouting shrubs and unburned shrubs were collected on consecutive days due to time constraints. We collected four young, fully expanded leaves, including the leaf used for gas exchange measurements, from the periphery and centre of each shrub for stable isotope analysis. Leaves from the first sampling date were collected 2 weeks after gas exchange measurements and all other leaves were collected immediately following gas exchange measurements. Leaves were stored in moist plastic bags within a cooler until returned to the laboratory. We measured leaf area using LEAFSCAN smartphone application (Anderson and Rosas-Anderson 2017). We then dried the leaf tissue at 60 °C for 72 h, and subsequently weighed each leaf for dry mass. We calculated leaf mass per area (LMA) as leaf dry mass (g)/leaf area (m^2^).

For each shrub, the four dried leaves from the periphery and centre were each combined and ground. We measured leaf nitrogen content per unit dry mass (N_mass_, mg g^−1^) and the stable carbon isotopic composition (δ ^13^C) of leaves at the Stable Isotope Mass Spectrometry Laboratory at Kansas State University. Total C and N of homogenized samples were determined following combustion using an Elementar vario Pyro cube coupled to an Elementar Vision mass spectrometer for isotope analysis. Isotopic abundance ratios were converted to *δ* notation using:


$$\begin{array}{l} \delta =\left[\frac{R_{sample}}{R_{standard}}\;-1\right]*1000 \end{array}$$ (1)

where *R* is the ratio of heavy to light isotopes for the sample and standard (Vienna-Pee-Dee Belemnite), respectively. Within-run and across-run variability of the laboratory working standard was <0.05 ‰. δ ^13^C was used as a proxy for integrated water use efficiency (WUE). Larger, enriched leaf δ ^13^C values indicate a higher integrated WUE (Farquhar *et al.* 1989). We expected trends to be similar for integrated WUE and iWUE (*A*/*g*_s_) with differences reflecting integrated WUE as a longer-term measurement incorporating daily WUE over the physiologically active life span of the leaf while iWUE is an instantaneous measurement.

N content per unit leaf area (N_area_, g m^−2^) was calculated as the N content of the homogenized leaf samples (g g^−1^) multiplied by the average leaf area (m^2^) for the centre and periphery of each shrub. Photosynthetic nitrogen use efficiency (PNUE) was calculated as:


$$\begin{array}{l} PNUE=\frac{A\;(\mu mol\;m^{-2}s^{-1})}{N_{area}(g\;m^{-2})} \end{array}$$ (2)

### Statistical analysis

Statistical analyses were performed in R V3.6.0 (R Core Team 2020). We used repeated measures type III ANOVA using the lmer function from the lme4 package (Bates 2015) and the ANOVA function from the car package (Fox and Weisburg 2019). For each analysis, pairwise comparisons were made using the emmeans package (Lenth 2020) with Tukey’s HSD adjustment. We ran three separate analyses to answer each of our three main questions:

(1) To assess how leaf physiology differed across ramet locations within the shrub, we analysed data from 2015 and 2018 separately since year of measurement had a different number of sampling dates and sampling locations within each shrub clone. We used location within clone (periphery to centre) as a fixed factor and sampling date (Day of Year, [DOY]) as a continuous predictor. Shrub ID was included as a random effect to account for repeated measures. We scaled DOY and shrub area to have a mean of 0 and a standard deviation of 1 so all numeric predictors had equal weight in the analyses (Greig-Smith 1983). We included shrub canopy area as a covariate, but it was not significant in any model (*P* > 0.05).
(2) To assess how leaf physiology differed between wet and dry years, we compared gas exchange and iWUE between 2015 (wet) and 2018 (dry). We used the same model as above and included year as a main effect, only comparing data from the common watershed (4B or unburned shrubs) and common sampling locations within each shrub (periphery and centre).
(3) We only used data from 2018 to assess differences in leaf physiology between resprouting shrubs (burned in 2018) and unburned shrubs. We used the same model as above and included fire as a main effect. For this model, we log transformed stomatal conductance (*g*_s_) to meet the assumptions of normality and homogeneity of variance.

## Results

### Effect of ramet location on leaf physiology depended on fire not year

**Figure 2.**
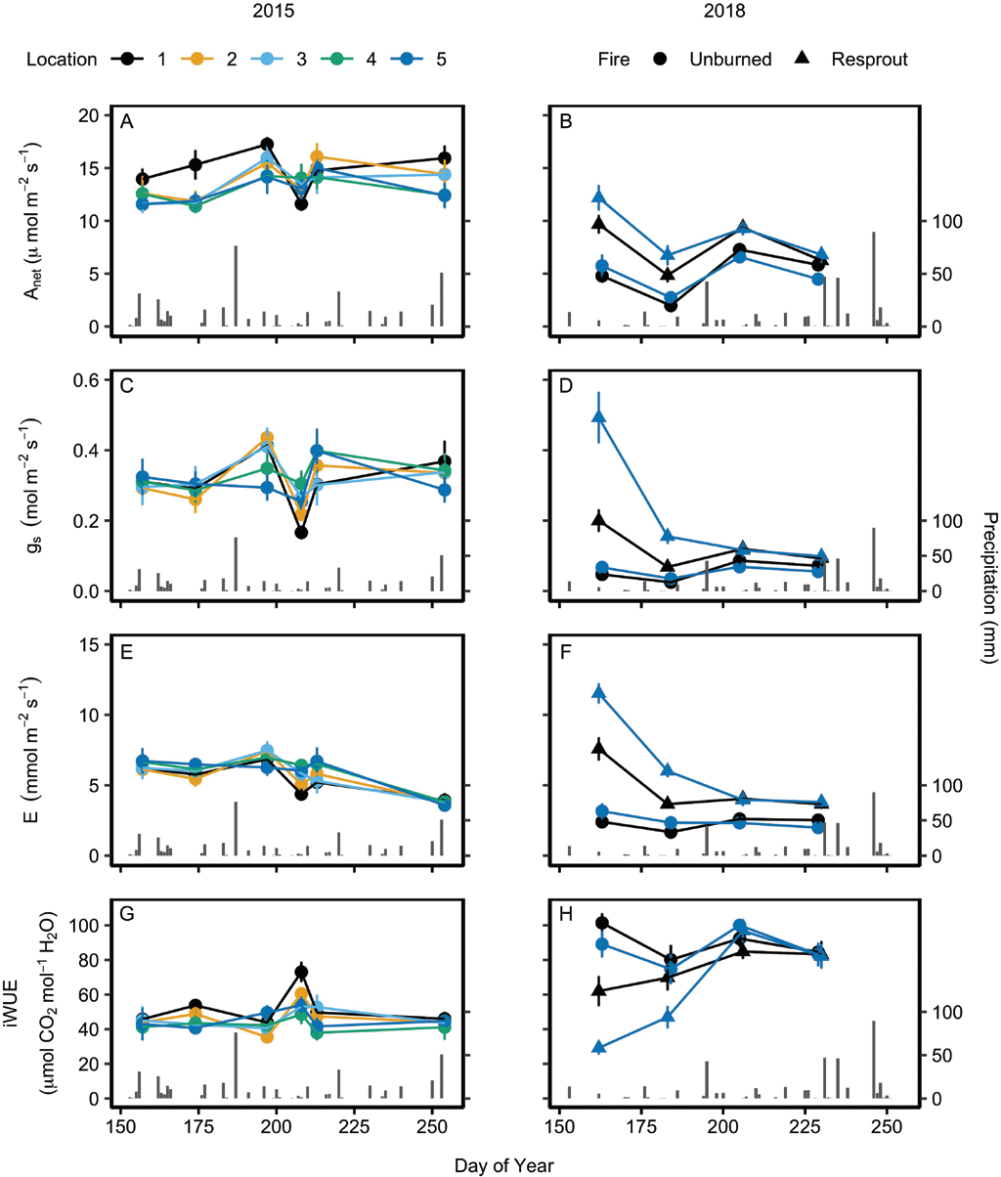
Mean (±1 SE) leaf gas exchange rates across various locations within *C. drummondii* clones during the 2015 and 2018 growing seasons, including net photosynthetic rate, *A*_net_ (A and B), stomatal conductance to vapour, *g*_s_ (C and D), transpiration rate, *E* (E and F) and intrinsic water use efficiency, iWUE (G and H). Locations 1 through 5 represent the outermost ramet to the centremost ramet of a shrub clone, respectively. For 2018, resprouting shrubs were shrubs recovering from prescribed fire (burned in 2018) and unburned shrubs were burned the previous year (burned in 2017). **See**Table 1 for repeated measures ANOVA statistics.

**Figure 3.**
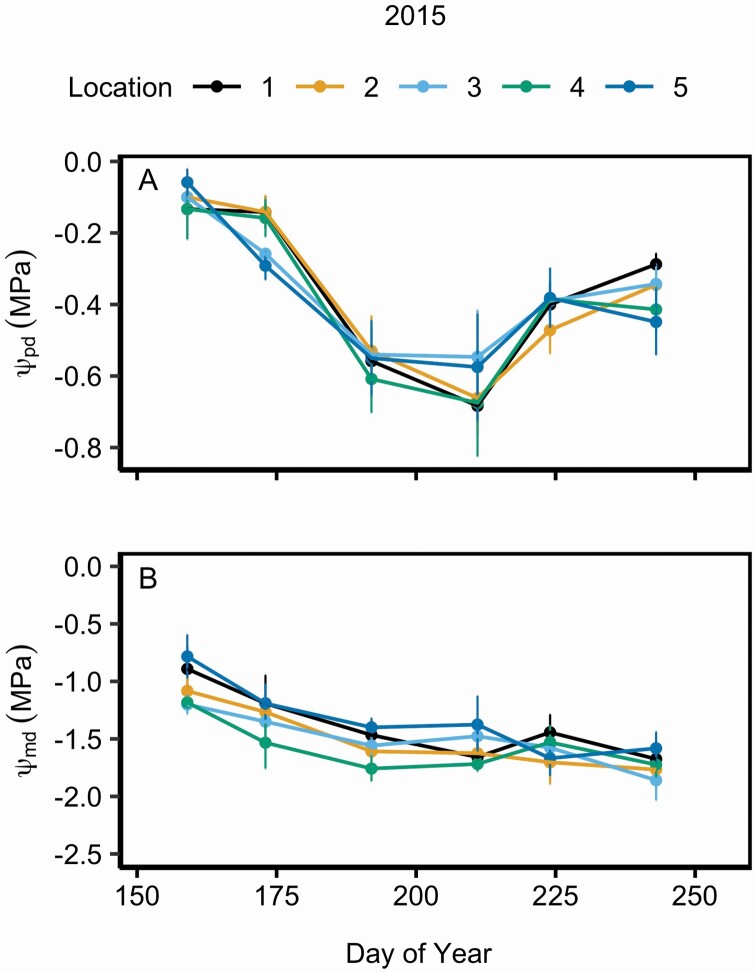
Mean (±1 SE) predawn, ψ _pd_ (A) and midday, ψ _md_ (B) water potentials across various locations within *C. drummondii* clones during 2015. Locations 1 through 5 represent the outermost ramet to the centremost ramet of a shrub clone, respectively. **See****Supporting Information—Table S5** for repeated measures ANOVA statistics.

Leaf physiology did not differ across ramet locations within unburned shrubs (Fig. 2; **see****Supporting Information—Tables S2–S5**). In 2015, photosynthetic rates (*A*_net_) slightly but significantly increased and transpiration rates (*E*) significantly decreased across the growing season (Table 1; Fig. 2A and E), but gas exchange rates did not differ among ramet locations within the shrubs (Table 1). Differences in average *A*_net_ were largest between the periphery (location 1: 14.81 ± 0.46 SEM µmol m^−2^ s^−1^) and centre (location 5: 13.01 ± 0.46 SEM µmol m^−2^ s^−1^) but these were not significant. On average, intrinsic water use efficiency (iWUE) was significantly higher on the periphery (location 1: 52.00 ± 2.25 SEM µmol mol^−1^) than near the centre (location 4: 42.55 ± 2.22 SEM µmol mol^−1^) of the shrub (*P* = 0.013; **see****Supporting Information—Table S2**). Leaf water potentials significantly differed throughout the growing season (*P* < 0.001; **see****Supporting Information—Table S5**), where ψ _pd_ was lowest during the middle of the season in July (DOY 192 and 211) and ψ _md_ decreased across the growing season (Fig. 3). Neither ψ _pd_ nor ψ _md_ differed among ramet locations within shrub clones.

**Table 1.** Type III ANOVA table of results for gas exchange measurements among ramet locations within *C. drummondii* shrubs in 2015 and 2018, including net photosynthetic rate (*A*_*net*_ µmol m^−2^ s^−1^), stomatal conductance (*g*_*s*_ mol m^−2^ s^−1^), transpiration (*E* mmol m^−2^ s^−1^), and intrinsic water use efficiency (iWUE µmol CO_2_ mol^−1^ H_2_O). Shown are *F*- and *P*-values for the fixed effects of ramet location within shrub, day of year (DOY), fire treatment, and their interactions. Shrub area was used as a covariate and was not included in the interactions. Bold *P*-values are statistically significant (α = 0.05).

Year	Predictor	*A*_*net*_		*g*_*s*_		*E*		iWUE	
		*F*	*P*	*F*	*P*	*F*	*P*	*F*	*P*
2015	Location	2.329	0.058	0.264	0.901	1.291	0.276	2.672	**0.034**
	DOY	6.873	**0.009**	1.399	0.239	49.337	**<0.001**	0.244	0.622
	Area	0.261	0.636	0.224	0.661	0.039	0.854	0.467	0.532
	Location × DOY	0.456	0.768	0.247	0.911	0.115	0.977	0.064	0.993
2018	Fire	39.600	**<0.001**	57.408	**<0.001**	94.979	**<0.001**	17.981	**<0.001**
	Location	1.109	0.293	5.973	**0.015**	13.099	**<0.001**	2.409	0.122
	DOY	0.560	0.455	7.577	**0.006**	83.988	**<0.001**	15.682	**<0.001**
	Area	0.335	0.566	0.506	0.481	0.217	0.644	0.021	0.886
	Fire × Location	2.628	0.106	7.791	**0.006**	10.852	**0.001**	1.089	0.298
	Fire × DOY	12.820	**<0.001**	59.781	**<0.001**	70.823	**<0.001**	22.117	**<0.001**
	Location × DOY	4.894	**0.028**	18.356	**<0.001**	24.258	**<0.001**	6.676	**0.010**
	Fire × Location × DOY	0.026	0.873	0.934	0.335	4.744	**0.030**	1.723	0.191

**Figure 4.**
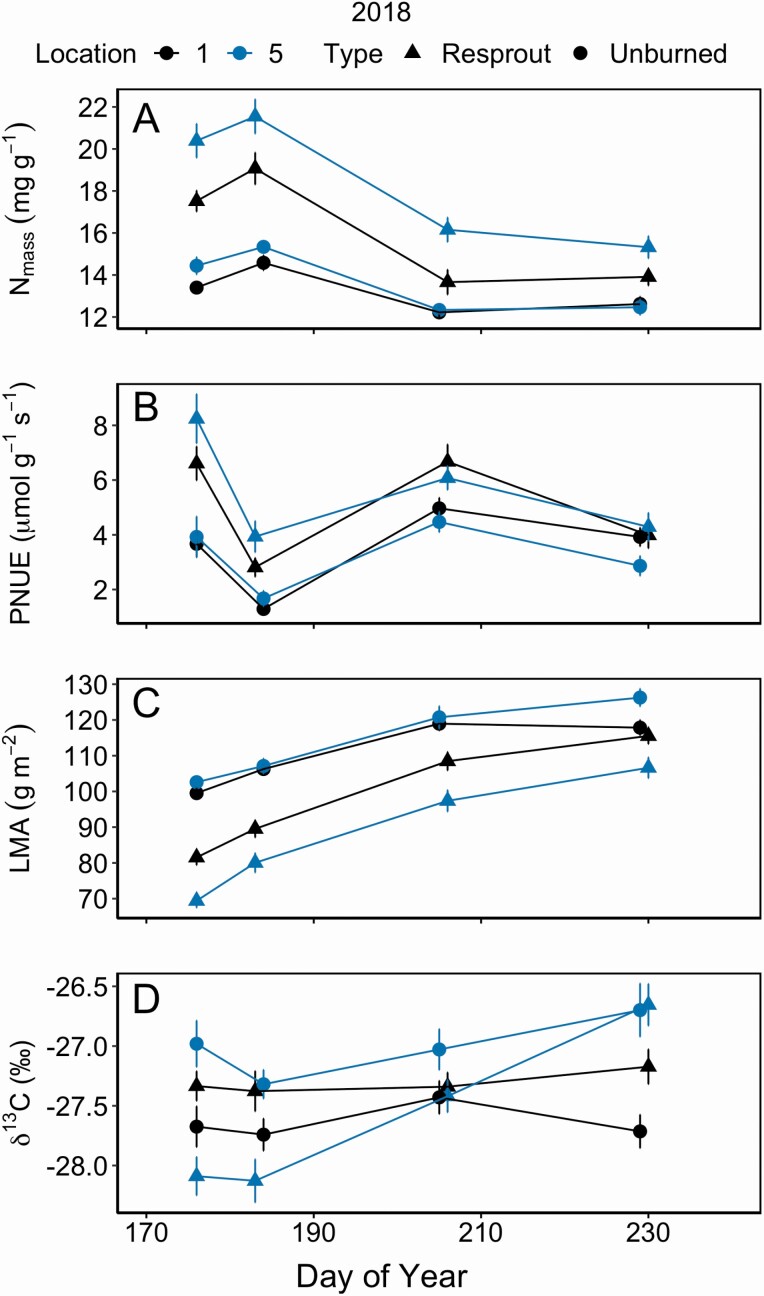
Mean (±1 SE) leaf traits between the periphery (location 1) and centre (location 5) of *C. drummondii* clones during 2018, including leaf nitrogen content, N_mass_ (A), photosynthetic nitrogen use efficiency, PNUE (B), leaf mass per area, LMA (C) and integrated water use efficiency, δ ^13^C (D). **See****Supporting Information—Table S6** for repeated measures ANOVA statistics.

In 2018, gas exchange rates were significantly different between the centre and periphery or resprouting shrubs **[see****Supporting Information—Table S2****]**. These differences were largest at the beginning of the growing season and diminished through time (Fig. 2B, D, F and H). On average, the centre of resprouting shrubs had 52 % greater *E* and almost 145 % greater *g*_s_ than the periphery during the first sampling period, but these differences decreased by the second sampling date (Fig. 2D and F). For unburned shrubs, there were no differences in gas exchange rates between the centre and periphery **[see****Supporting Information—Table S2****]**. In resprouting shrubs, leaves from the centre of the clone had significantly higher N_mass_ (*P* < 0.001) and lower LMA (*P* < 0.001) than leaves from the periphery of the clone (Fig. 4A and C; **see****Supporting Information—Table S6**). Integrated WUE (δ ^13^C) showed a significant 3-way interaction among burn treatment, location within shrub, and DOY (Fig. 4D; **see****Supporting Information—Table S6**; *P* = 0.004). δ ^13^C values had low variability across the growing season for leaves from the periphery of resprouting and unburned shrubs (standard deviation: 0.610 and 0.653, respectively) while δ ^13^C slightly but significantly increased in leaves from the centre of the shrub clones over time (DOY and location interaction: *P* < 0.001).

### Leaf physiology of unburned shrubs differed between wet and dry years

On average, the centre and periphery of unburned shrubs in 2015 had significantly higher gas exchange rates (*A*_net_, *E* and *g*_s_) and lower iWUE than unburned shrubs in 2018 (Fig. 2A–H; Table 2).

**Table 2.** Type III ANOVA table of results comparing gas exchange measurements between center and periphery ramet locations within *C. drummondii* shrubs in 2015 and 2018, including net photosynthetic rate (*A*_*net*_ µmol m^−2^ s^−1^), stomatal conductance (*g*_*s*_ mol m^−2^ s^−1^), transpiration (*E* mmol m^−2^ s^−1^), and intrinsic water use efficiency (iWUE µmol CO_2_ mol^−1^ H_2_O). Shown are *F*- and *P*-values for the fixed effects of ramet location within shrub, day of year (DOY), fire treatment, and their interactions. Shrub area was used as a covariate and was not included in the interactions. Bold *P*-values are statistically significant (α = 0.05).

Comparison	Predictor	*A*_*net*_		*g*_*s*_		*E*		iWUE	
		*F*	*P*	*F*	*P*	*F*	*P*	*F*	*P*
2015 vs. 2018	Year	295.694	**<0.001**	439.689	**<0.001**	189.625	**<0.001**	75.599	**<0.001**
	Location	6.064	**0.015**	0.003	0.953	3.867	**0.051**	1.464	0.228
	DOY	5.607	**0.019**	1.490	0.223	24.656	**<0.001**	0.063	0.801
	Area	0.163	0.692	1.356	0.267	0.008	0.929	0.0002	0.989
	Year × Location	3.668	0.057	0.078	0.780	2.460	0.118	0.493	0.483
	Year × DOY	0.670	0.414	0.466	0.495	15.056	**<0.001**	0.389	0.533
	Location × DOY	1.286	0.258	2.299	0.131	5.122	**0.025**	0.686	0.408
	Year × Location × DOY	2.221	0.138	0.010	0.919	2.135	0.145	0.398	0.528

### Leaf physiology differed between resprouts and unburned shrubs in 2018

In 2018, resprouting shrubs had on average 60 % higher *A*_net_, 165 % higher *g*_s_ and 122 % higher *E* than unburned shrubs (Fig. 2B, D and F). The interaction between DOY and fire was significant (Table 1) for all gas exchange variables due to resprouts having higher rates at the beginning of the season. Resprouting and unburned shrubs exhibited similar trends in *A*_net_ following precipitation events. Average *A*_net_ rates on the second sampling date were nearly half the rates measured on the first sampling date and increased again after the site received multiple days with rainfall (Fig. 2B). Resprouting shrubs had significantly lower iWUE (Table 1) than the unburned shrubs, with greater differences between fire treatments at the beginning of the season than the end of the season (Fig. 2H). Leaves from resprouting shrubs had significantly higher N_mass_ and lower LMA than unburned shrubs (*P* < 0.001; Fig. 4A and C). Integrated WUE followed similar trends, as resprouting shrubs had lower δ ^13^C in the centre of the clones at the beginning of the season than unburned shrubs and this difference decreased over time (Fig. 4D). PNUE was significantly higher in resprouts than unburned shrubs (*P* < 0.001; Fig. 4B).

## Discussion

Clonal growth is the dominant growth strategy in grasslands (Benson and Hartnett 2006) and is associated with increased establishment, growth and survival. Although clonal shrubs are increasing in abundance throughout grasslands and savannas globally, we currently lack an understanding of how physiological processes differ among ramet locations within shrub clones or shift in response to disturbance. In this study, we investigated if leaf-level physiology differs among ramet locations within a common clonal shrub known to exhibit water transfer among ramets and if these patterns are altered by fire or drought. Gas exchange rates did not differ among ramet locations within unburned *C. drummondii* during both wet and dry years. However, leaf physiology differed among ramets within resprouting shrubs shortly after a fire, and gas exchange rates of recently burned shrubs were higher than that of unburned shrubs. These results suggest that physiological integration within this clonal shrub maintains similar intra-clonal gas exchange rates in both wet and dry years, and that increased gas exchange rates after fire can contribute to recovery of above-ground biomass, particularly during an extremely dry growing season.

### Effect of ramet location on leaf physiology depended on fire not year

Resource transfer among ramets can increase the physiological uniformity, productivity and competitive ability of clonal shrubs (reviewed in Song *et al.* 2013). Previous work has shown that *C. drummondii* transfers resources within individuals, where younger ramets on the clone periphery are reliant on water derived from older ramets in the centre (Ratajczak *et al.* 2011; Killian 2012). Here, we found that leaf-level physiology did not differ across different ramet locations within unburned *C. drummondii* shrubs, suggesting that clonal integration among ramets likely buffers differences in gas exchange rates among ramet locations throughout a clone (Fig. 2). Maintaining high carbon assimilation rates throughout a clone may be beneficial for survival of the entire individual in a variable environment and may also increase shrub size by promoting clonal lateral growth (Götmark *et al.* 2016). While it is unknown how long *C. drummondii* ramets remain connected, we found no differences in leaf-level physiology across different sized clones (Table 1), suggesting that this clonal shrub retains physiological integration at large sizes (at least up to 347 m^2^).

Lower LMA and higher N_mass_ of ramets in the centre compared with the periphery of resprouting shrubs likely enabled the high photosynthetic rates observed early in the growing season, which could potentially facilitate carbon movement within the shrub and allow for both rapid regrowth in peripheral leaves and increased lateral spread of the clone (McCarron and Knapp 2003). LMA indicates the carbon invested per light-capturing area and can represent a trade-off between leaf longevity and carbon gain, where species with lower LMA have thinner, ‘cheaper’ leaves that are more efficient at acquiring carbon (Wright *et al.* 2004). Species with low LMA also tend to have higher foliar N content, which facilitates the higher photosynthetic rates observed in the leaves of resprouts (Evans 1989; Poorter *et al.* 2009). Additionally, greater leaf area in the centre of the shrub would support higher area-based transpiration where the root:shoot ratio is the highest, which may enable more efficient water transfer throughout the clone.

### Leaf physiology of unburned shrubs differed between wet and dry years

The summer of 2018 was one of the driest and hottest on record at KPBS leading to lower gas exchange rates in 2018 than 2015. Although gas exchange rates were lower overall during the dry year, *g*_s_ did not differ significantly in unburned shrubs within growing seasons, suggesting that *C. drummondii* may exert loose stomatal control over leaf water status (i.e. exhibit ‘anisohydric’ stomatal behaviour; Tardieu and Simonneau 1998). For instance, in 2015, average *g*_s_ between the initial and final sampling periods differed by ~8 %, despite intra-annual variation in precipitation, while ψ _md_ declined by ~68 % across the growing season (Figs 2C–3B). Although leaf water potential was not measured in 2018, stomata were similarly insensitive to intra-annual variation in microclimate during the drought. *Cornus drummondii* is deep-rooted and has access to a consistent water source (Ratajczak *et al.* 2011; Nippert *et al.* 2013), which likely allows leaves to maintain similar *g*_s_ throughout the growing season despite intra-annual fluctuations in precipitation, atmospheric temperature or vapour pressure deficit (O’Keefe *et al.* 2020). However, if deep water stores are depleted during extreme or prolonged future drought, *C. drummondii* may experience a leaf water potential threshold that induces stomatal closure (Klein 2014; Meinzer *et al.* 2016) and overall gas exchange rates may decline. Thus, the maintenance of similar *g*_s_ and *A*_net_ throughout a growing season may benefit *C. drummondii* during years of average or above average precipitation but might negatively impact the functioning of this species in a warmer, drier climate.

### Leaf physiology differed between resprouts and unburned shrubs in 2018

Increased carbon gain could facilitate more rapid plant recovery from fire to compete with vigorous grass growth, recover lost above-ground biomass and replenish below-ground reserves used for resprouting (Landhausser and Lieffer 2002; Schutz *et al.* 2009). The high gas exchange rates we observed in resprouting shrubs is consistent with other studies (DeSouza *et al.* 1986; McCarron and Knapp 2003; Goorman *et al.* 2011) and likely due to a combination of factors, including increased leaf N content and root:shoot ratio. Increased leaf N after fire supports the transient maxima hypothesis, which posits the accumulation of N in infrequently burned grasslands is readily taken up by resprouting plants after fire due to a transient release from N and light limitation (Seastedt and Knapp 1993; Blair 1997; McCarron and Knapp 2003). High leaf N content is positively correlated with photosynthetic capacity as the majority of leaf N is used to build photosynthetic machinery (Field and Mooney 1986; Evans 1989), resulting in resprouts that have higher photosynthetic and growth rates than shrubs that were not recently burned (DeSouza *et al.* 1986; Castell *et al.* 1994; Fleck *et al.* 1998; Goorman *et al.* 2011).

In addition to increased leaf N_mass_, the higher gas exchange rates observed in the resprouts may also be due to a greater root:shoot ratio resulting in increased soil water and nutrient availability for individual leaves due to reduced canopy size (Peña-Rojas *et al.* 2004; Goorman *et al.* 2011). Deep roots provide a consistent water source allowing new above-ground growth to exert high transpiration rates at the beginning of the season. Increased transpiration rates in resprouting shrubs can buffer leaf microclimate by increasing latent heat and decreasing leaf temperature after fire (Gates 1968; Michaletz 2015). For instance, O’Connor (2019) showed that leaf temperature was lower than the ambient air temperature for recently burned, but not unburned *C. drummondii*, and was likely due to the increased stomatal conductance and transpiration rates observed during resprouting. This suggests the deep roots of *C. drummondii* likely have multiple impacts on leaf carbon, water and thermal energy budgets following disturbance by favouring latent over sensible heat exchange and creating a more favourable microclimate for photosynthesis during regrowth. Increased latent heat may be particularly important for regrowth during drought, like 2018, when air temperatures near the soil surface are high due to decreased albedo after fire in conjunction with warmer than average air temperatures (Bremer and Ham 1999).

Increased gas exchange rates and lower iWUE observed in recently burned shrubs correspond with changes in leaf structure associated with higher growth rates (i.e. LMA). As described previously, resprouts had lower LMA, higher N_mass_, and higher PNUE than unburned shrubs, suggesting that resprouting shrubs invest in structurally ‘cheap’ leaves with high N to maximize carbon gain and increase growth rates after fire. These post-fire physiological traits are attributes that help clonal shrubs respond to disturbances, increase competitive success with grasses, and ultimately promote long-term persistence and expansion in highly disturbed environments.

### Implications

Clonal shrub encroachment can have significant ecosystem consequences for tallgrass prairie. In mesic grasslands, shrub encroachment often increases above-ground net primary productivity (Lett *et al.* 2004; Knapp *et al.* 2008) and soil organic carbon (Li *et al.* 2016). Deep-rooted shrubs may also shift grassland ecohydrology by using deep soil water relative to herbaceous species (Nippert and Knapp 2007) and by transpiring more water than other co-occurring species (O’Keefe *et al.* 2020). Considering grasslands will likely become warmer and drier as climate change progresses, a shift in vegetation cover from herbaceous species to deep-rooted woody shrubs may deplete deep water stores and increase evapotranspiration, altering ecosystem water flux (Huxman 2005; Scott *et al.* 2014; Logan and Brunsell 2015; Honda and Durigan 2016; Acharya *et al.* 2017; O’Keefe *et al.* 2020).

Given the global increase in shrub encroachment, as well as the numerous ecosystem consequences associated with this phenomenon, there is current interest in modelling changes in grass-shrub cover, as well as the resulting shifts in ecosystem carbon and water budgets, within herbaceous ecosystems (O’Donnell and Caylor 2012; Qiao *et al.* 2015; Scheiner-McGraw *et al.* 2020). Traditionally, ecosystem models that predict the distribution of vegetation types (i.e. dynamic global vegetation models), as well as land-surface models that estimate the exchange of mass and energy within the Earth-atmosphere system, group plant species into coarse plant functional type groups based on life history traits (e.g. deciduous or evergreen) and morphological plant traits (e.g. leaf life span, leaf mass per area, leaf nitrogen content; Fisher *et al.* 2015). More recent efforts to improve model predictions of vegetation dynamics have incorporated additional biological complexity to models (e.g. by parameterizing plant hydraulic traits; Christoffersen *et al.* 2016), but still do not account for potential heterogeneity in physiological functioning within clones. Consequently, if clonal shrubs do exhibit heterogeneity in functioning, models that represent these species as a single functional type based on coarsely defined plant traits may produce inaccurate assessments of shrub encroachment dynamics.

Our data show that leaf physiological traits do not differ substantially among ramet locations within *C. drummondii* clones at a given point during the summer, nor do these patterns shift in response to typical intra-annual variability in microclimate or in response to drought. These results are important because they suggest that single trait values of shrub clones measured within the growing season may be sufficient for parameterizing *C. drummondii* in vegetation models that aim to understand shrub encroachment under different climate scenarios. However, we did find intra-clonal physiological differences shortly after fire, as well as substantial differences in physiology between recently burned and unburned shrubs. These results indicate that fire dynamics should be considered when modelling shrub encroachment dynamics (e.g. using the SPITFIRE model; Thonicke *et al.* 2010), and that trait parameters may require additional complexity in the context of fire. Future work should conduct detailed investigations of intra-clonal physiology for other shrubs that are expanding across grasslands and savannas in order to improve predictions of vegetation cover and ecosystem functioning in a changing climate.

## Supporting Information

The following additional information is available in the online version of this article—

**Table S1**. Sampling dates for photosynthetic gas exchange and water potential in 2015 and 2018.

**Table S2.** Tukey’s pairwise comparisons for gas exchange rates among ramet locations within clonal shrubs in 2015 and 2018.

**Table S3.** Tukey’s pairwise comparisons for gas exchange rates between unburned shrubs in 2015 and 2018.

**Table S4.** 95% confidence interval of gas exchange rates for each ramet location in 2015 and 2018.

**Table S5.** Type III ANOVA table of results for predawn and midday water potential among ramet locations with *C. drummondii* shrubs in 2015.

**Table S6.** Type III ANOVA table of results for leaf traits among ramet locations within *C. drummondii* shrubs in 2018.

**Figure S1.** Cumulative precipitation, mean daily temperature, and vapor pressure deficit from April 1–September 30 in 2015 and 2018.

**File S1.** R code for gas exchange data.

**File S2.** R code for water potential data.

**File S3.** R code for leaf trait data.

plab037_suppl_Supplementary_Materials

## Data Availability

Data and metadata are available on the Long Term Ecological Research Network Data Portal https://portal.edirepository.org.

## Notes

**Form & Function. Chief Editor:** Kate McCulloh
