# Supplementary material for: Spatio-temporal differences in leaf physiology are associated with fire, not drought, in a clonally integrated shrub
Source: AoB Plants. 2021 Jun 9;13(4):plab037. doi: 10.1093/aobpla/plab037 (PMC8317628; doi:10.1093/aobpla/plab037)
Supplement: plab037_suppl_Supplementary_Materials [file plab037_suppl_supplementary_materials.zip › plab037_suppl_Wedel_SupportingInfo_Revised.pdf]

Table S1. Sampling dates for photosynthetic gas exchange and water potential in 2015 and 2018. Watershed refers to two watersheds on Konza Prairie with a 4-year prescribed burn frequency. 4B was burned in 2017 and 4A was burned in 2018. Day of year refers to the number of days since January 1 (day 1).

Year	Watershed	Measurement	Month	Day of Year
2015	4B	gas exchange	June	157
				174
			July	197
				208
			August	213
			September	254
2015	4B	water potential	June	159
				173
			July	192
				211
			August	224
				243
2018	4A	gas exchange	June	162
			July	183
				206
			August	230
2018	4B	gas exchange	June	163
			July	184
				205
			August	229

Table S2. Tukey's pairwise comparisons for gas exchange rates among ramet locations within clonal shrubs in 2015 and 2018, including instantaneous net photosynthetic rate (A_{net}), stomatal conductance (g_s), transpiration (E), and intrinsic water use efficiency (iWUE). Contrast refers to the ramet locations within clonal shrubs, where location 1 is the outermost ramet on the periphery and location 5 is the innermost ramet in the center of each shrub. All shrubs were located in areas with a 4-yr burn frequency. 'Unburned' refers to shrubs that did not experience fire the year of measurements, while 'resprout' refers to shrubs that had resprouted after fire in April of 2018. Shown are P-values for each comparison. Bolded P-values are statistically significant (α=0.05).

Year	Fire	Contrast	A_{net}	g_s	E	iWUE
2015	unburned	1-2	0.651	0.998	0.968	0.389
		1-3	0.327	0.993	0.797	0.313
		1-4	0.097	0.891	0.263	**0.013**
		1-5	0.059	1.000	0.455	0.202
		2-3	0.984	1.000	0.989	0.999
		2-4	0.790	0.973	0.645	0.608
		2-5	0.674	0.992	0.841	0.995
		3-4	0.974	0.987	0.897	0.695
		3-5	0.929	0.996	0.981	0.999
		4-5	0.999	0.914	0.996	0.829
2018	resprout	1-5	**0.050**	**< 0.001**	**< 0.001**	0.052
2018	unburned	1-5	0.732	0.867	0.772	0.701

Table S3. Tukey's pairwise comparisons for gas exchange rates between unburned shrubs in 2015 and 2018, including instantaneous net photosynthetic rate (A_{net}), stomatal conductance (g_s), transpiration (E), and intrinsic water use efficiency (iWUE). All shrubs were located in the same watershed with a 4-yr burn frequency. Shown are P-values for each comparison. Bolded P-values are statistically significant (α=0.05).

Ramet Location	Fire	Contrast	A_{net}	g_s	E	iWUE
1	unburned	2015 - 2018	**< 0.001**	**< 0.001**	**<0.001**	**<0.001**
5	unburned	2015 - 2018	**< 0.001**	**< 0.001**	**<0.001**	**<0.001**

Table S4. 95% confidence interval of gas exchange rates for each ramet location in 2015 and 2018 including instantaneous net photosynthetic rate (*A_{net}*), stomatal conductance (*g_s*), transpiration (*E*), and intrinsic water use efficiency (iWUE). Ramet location refers to the location of ramets within each clonal shrub, where 1 is the outermost ramet on the periphery and 5 is the innermost ramet in the center of each shrub. All shrubs were located in areas with a 4-yr burn frequency. ‘Unburned’ refers to shrubs that did not experience fire the year of measurements, while ‘resprout’ refers to shrubs that had resprouted after fire in April of 2018.

Year	Fire	Ramet location	A_{net}	g_s	E	iWUE
2015	unburned	1	(13.8, 15.8)	(0.266, 0.351)	(4.77, 6.00)	(47.1, 56.0)
		2	(12.9, 14.9)	(0.274, 0.359)	(4.99, 6.22)	(41.8, 51.6)
		3	(12.5, 14.6)	(0.277, 0.362)	(5.15, 6.39)	(41.3, 51.2)
		4	(12.1, 14.2)	(0.290, 0.374)	(5.46, 6.70)	(37.5, 47.3)
		5	(12.0, 14.0)	(0.268, 0.353)	(5.35, 6.58)	(40.6, 50.5)
2018	resprout	1	(6.53, 8.21)	(0.075, 0.107)	(3.98, 4.94)	(69.3, 83.1)
		5	(7.61, 9.29)	(0.109, 0.154)	(5.37, 6.33)	(60.2, 73.9)
2018	unburned	1	(4.21, 5.93)	(0.040, 0.058)	(1.81, 2.78)	(81.6, 95.8)
		5	(4.02, 5.73)	(0.040, 0.057)	(1.89, 2.86)	(79.8, 93.9)

Table S5. Type III ANOVA table of results for predawn and midday water potential (ψ_{pd} and ψ_{md}, respectively) among ramet locations within *C. drummondii* shrubs in 2015. Shown are F- and P-values for the fixed effects of ramet location within shrub, day of year (DOY), and their interactions. Bolded P-values are statistically significant (α=0.05).

Year	Predictor	ψ_{pd}		ψ_{md}	
		F	P	F	P
2015	Location	0.115	0.977	2.434	**0.049**
	DOY	32.651	**<0.001**	63.894	**<0.001**
	Area	0.293	0.617	0.653	0.464
	Location*DOY	0.183	0.947	0.898	0.467

Table S6. Type III ANOVA table of results for leaf traits among ramet locations within *C. drummondii* shrubs in 2018, including nitrogen content (N_{mass}), photosynthetic nitrogen use efficiency (PNUE), leaf mass per area (LMA), and integrated water use efficiency ($\delta^{13}C$). Shown are F- and P-values for the fixed effects of ramet location within shrub (center or periphery), day of year (DOY), fire treatment (unburned or resprouting shrubs), and their interactions. Bolded P-values are statistically significant (α=0.05).

Year	Predictor	N_{mass}		PNUE		LMA		$\delta^{13}C$	
		F	P	F	P	F	P	F	P
2018	Fire	76.688	**<0.001**	41.048	**<0.001**	57.85	**<0.001**	0.466	0.499
	Location	32.65	**<0.001**	0.422	0.517	15.284	**<0.001**	8.226	**0.004**
	DOY	155.039	**<0.001**	0.002	0.967	621.291	**<0.001**	42.843	**<0.001**
	Area	0.418	0.522	0.269	0.607	0.473	0.496	1.383	0.247
	Fire*Location	15.202	**<0.001**	1.745	0.188	62.367	**<0.001**	50.547	**<0.001**
	Fire*DOY	30.305	**<0.001**	7.307	**0.007**	33.824	**<0.001**	13.786	**<0.001**
	Location*DOY	3.983	**0.047**	3.951	**0.048**	3.092	0.08	27.436	**<0.001**
	Fire*Location*DOY	0.001	0.974	0.009	0.925	0.772	0.38	8.61	**0.004**

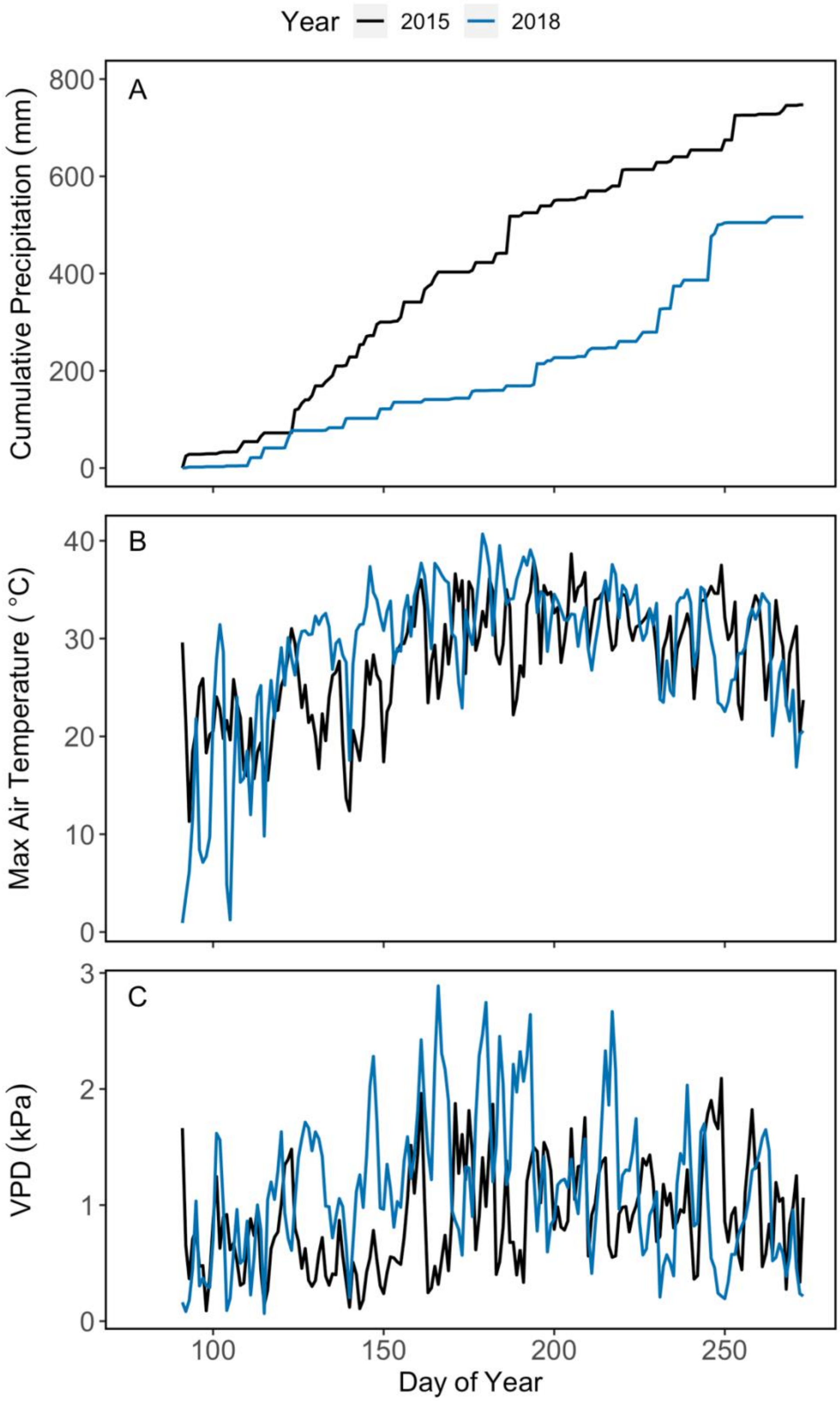

Figure S1. Cumulative precipitation (mm) mean daily temperature (°C), and vapor pressure deficit (kPA) from April 1 – September 30 in 2015 (black) and 2018 (blue).
